# Impact of repetitive transcranial magnetic stimulation on clinical and cognitive outcomes, and brain-derived neurotrophic factor levels in treatment-resistant depression

**DOI:** 10.3389/fpsyt.2025.1584673

**Published:** 2025-04-08

**Authors:** Enrico Ginelli, Lucia Sanna, Pasquale Paribello, Ulker Isayeva, Giorgio Corona, Clement C. Zai, Daniela Manca, Maria Novella Iaselli, Roberto Collu, Federica Pinna, Maria Scherma, Mirko Manchia, Paola Fadda, Bernardo Carpiniello

**Affiliations:** ^1^ Section of Psychiatry, Department of Medical Sciences and Public Health, University of Cagliari, Cagliari, Italy; ^2^ Unit of Clinical Psychiatry, University Hospital Agency of Cagliari, Cagliari, Italy; ^3^ Center for Transcranial Magnetic Stimulation, Cagliari, Italy; ^4^ Tanenbaum Centre for Pharmacogenetics, Campbell Family Mental Health Research Institute, Centre for Addiction and Mental Health, Toronto, ON, Canada; ^5^ Department of Psychiatry, Institute of Medical Science, Laboratory Medicine and Pathobiology, University of Toronto, Toronto, ON, Canada; ^6^ Department of Biomedical Sciences, Division of Neuroscience and Clinical Pharmacology, University of Cagliari, Cagliari, Italy; ^7^ Centro Nazionale delle Ricerche (CNR) Institute of Neuroscience - Cagliari, National Research Council, Cagliari, Italy; ^8^ Department of Pharmacology, Dalhousie University, Halifax, NS, Canada

**Keywords:** rTMS, depression, treatment-resistance, BDNF, suicidality, neuroplasticity

## Abstract

**Introduction:**

Treatment-resistant depression (TRD) affects approximately 30% of patients with major depressive disorder (MDD), for whom effective treatment options are limited. Repetitive transcranial magnetic stimulation (rTMS) has shown efficacy in alleviating depressive symptoms in TRD. However, it remains unclear if these improvements are driven or mediated by changes in cognitive function or biological markers, such as brain-derived neurotrophic factor (BDNF).

**Methods:**

This study examines the effects of rTMS on depressive symptoms, cognition, and BDNF levels, as well as the potential moderating role of lifetime suicidal attempts (LSA) on cognition and the predictive value of baseline BDNF for clinical outcomes. Twenty-five TRD patients were included, with 13 in the rTMS treatment group (receiving 20 sessions of rTMS over four weeks) and 12 as control group. Depression severity, cognitive function (Mini-Mental State Examination, Verbal Fluency, Digit Span), and serum BDNF levels were measured pre- and post-treatment. Mixed-effects linear regression models assessed clinical and biological associations.

**Results:**

rTMS significantly reduced HAM-D (p < 0.001) and CGI (p < 0.001) scores compared to controls. Cognitive performance improved significantly in MMSE (p = 0.049) and Digit Span (p = 0.04), with no significant changes in BDNF levels (p = 0.39). LSA did not moderate cognitive outcomes, and baseline BDNF did not predict clinical improvement (p = 0.68).

**Discussion:**

rTMS reduced depressive symptoms in TRD patients, with modest cognitive benefits. Baseline BDNF did not predict outcomes, though the lack of significant change suggests complex neuroplastic responses. Future studies should include larger samples and refined biomarker assessments.

## Introduction

Major depressive disorder (MDD) is a severe, recurrent and often chronic psychiatric disorder affecting over 300 million people and about 5% of the general population (1). Despite continuous research efforts, its neurobiological underpinnings remain partly unexplained (2). Treatment-resistant depression (TRD) is a severe occurrence affecting a substantial portion of individuals diagnosed with MDD. Indeed, approximately 30% of patients with MDD receiving treatment meet the criteria for TRD (3), underscoring the need for more effective interventions. In fact, current interventions often fail to achieve adequate symptom remission (4). Among alternative treatment options, repetitive transcranial magnetic stimulation (rTMS), a non-invasive brain stimulation technique has garnered increasing attention for its therapeutic potential. Over the past few decades, rTMS has been shown to have a positive impact on depressive symptoms, making it a critical tool for managing TRD cases (5).

While the primary efficacy of rTMS in alleviating depressive symptoms is well-documented (6), its broader impact on cognitive functioning and biological markers, such as brain-derived neurotrophic factor (BDNF), remains a subject of ongoing investigation (7, 8). ​Beyond its effects on neuroplasticity, rTMS influences neurotransmitter systems implicated in depression. Some studies indicate that rTMS can selectively alter levels of gamma-aminobutyric acid (GABA) and glutamate (9, 10). These neurotransmitters play critical roles in mood regulation, and their modulation by rTMS may contribute to its therapeutic effects in reducing depressive symptoms.​ Cognitive deficits are common clinical manifestations of MDD (11), with data showing that the severity of depressive symptoms, rather than the specific diagnosis, is negatively correlated with cognitive performance (12). In particular, impairment in domains such as verbal fluency, working memory, and executive functions, is very common in individuals with MDD (13). However, the relationship between these cognitive impairments and rTMS-induced changes is not clearly understood. Some studies suggest that rTMS may positively affect cognitive functioning, but the magnitude and consistency of these effects vary widely (14, 15). Our study aims to further explore this relationship by assessing the impact of rTMS on a range of cognitive measures in TRD patients, including those assessed with the Mini-Mental State Examination (MMSE), the Verbal Fluency Test (VF) including its Categorical Fluency sub-test, and the Digit Span Test, from the Wechsler battery (16).

In addition to its effects on depressive symptoms and cognitive performance, rTMS has been hypothesized to influence neuroplasticity through the modulation of BDNF levels. BDNF is a critical protein involved in neuronal survival, growth, and synaptic plasticity (17). BDNF has emerged as a potential biomarker for depression, with lower serum BDNF levels consistently reported in depressed patients compared to healthy controls (18). Some studies have demonstrated an increase in BDNF levels following rTMS treatment (7), while others have reported no significant change or even a decrease in BDNF concentrations post-treatment (8, 19). Although several studies, summarized by this recent meta-analysis (20), casted doubts on the validity of BDNF as a predictor of treatment in TRD by itself, we sought to identify meaningful clinical implications by taking into account its role. The present study seeks to clarify this inconsistency by examining the serum BDNF levels before and after rTMS treatment in a sample of TRD patients.

Previous research has also identified a link between lifetime suicide attempts (LSA) and both cognitive deficits and altered BDNF levels, with suicide attempters often exhibiting worse cognitive performance and lower BDNF concentrations than non-attempters (21, 22). However, few studies have explored the interaction between LSA, cognitive functioning, and BDNF levels in the context of rTMS treatment. We hypothesize that there might be an interaction between LSA, cognitive function, and rTMS, in moderating the cognitive improvements in a negative way. Thus, this study has a threefold aim. First, we assessed the impact of rTMS treatment on depressive symptoms, cognitive performance, and BDNF levels in patients with TRD. Secondly, we tested the hypothesis of LSA having a moderating effect on cognitive improvements obtainable with rTMS in TRD patients. Lastly, we tested the hypothesis of baseline BDNF levels as a possible predictor of treatment outcome with rTMS on TRD patients. By combining these three analyses in our design, we sought to bring a better understanding of rTMS’s therapeutic potential and its underlying mechanisms in TRD.

## Materials and methods

### Study design and setting

This is a prospective, open-label, controlled study aimed at better understanding the impact of rTMS on TRD patients. The study was conducted at the University of Cagliari, within a specialized outpatient psychiatric clinic. All the patients involved in the study were enrolled from the clinic internal database, based on their clinical characteristics. The study was approved by the local Ethics Committee along with the primary analysis. All participants were required to sign an informed consent form before their involvement in the study. After identifying the 25 participants, we proceeded by splitting them into a control group (N=12) that received no treatment with rTMS and a treatment group (N=13) that received the rTMS treatment. The treatment group received the rTMS treatment consisting of 20 sessions in the span of 4 weeks. Participants in the control group continued to receive their standard pharmacological treatment, which was kept unchanged throughout the study period to ensure consistency and minimize potential confounding factors. No additional interventions were provided to the control group, and they did not undergo any form of rTMS, whether active or sham, and continued their treatment throughout the study. This approach was chosen to maintain ethical standards while isolating the effects of rTMS in the treatment group. The lack of sham procedure must be considered a limitation for this study and will be discussed in that section. We tested all our participants one week before the treatment (T_0_) and one week after administering rTMS to the treatment group (T_1_). At both timepoints, we administered the same battery of tests and collected blood samples to measure serum BDNF levels. Following data collection, we conducted statistical analyses using the appropriate statistical software to process our findings.

### Participants

This study was designed as an exploratory investigation of the effects of rTMS on TRD. As such, no formal *a priori* power analysis was conducted. Twenty-five patients (mean age 61.2 years (SD = 9.58), with a female-to-male ratio of 16:9) with TRD were enrolled based on their diagnoses, medical records, and recent clinical interviews with specialized staff at our clinic. Individuals were eligible for inclusion if they were: between 18 and 70 years of age, had a primary diagnosis of MDD, as defined by the DSM-5 (23), with a significant depression severity, defined with a score greater than 17 on the 21-item Hamilton Rating Scale for Depression (HAM-D 21) (24). Additionally, participants were required to have an active episode of TRD, characterized by an inadequate response to at least two different antidepressants, from two different classes of antidepressant medications. These treatments had to have been administered at standard dosages for a minimum of six weeks each within the past five years, corresponding to Stage II of the Thase and Rush staging model (25). These assessments were conducted by certified professionals, through interviews and clinical records search.

Exclusion criteria were established to rule out confounding factors that could interfere with the study’s outcomes. Participants were excluded if they exhibited progressive cognitive deterioration with a probable diagnosis of dementia or had a diagnosis of intellectual disability. Individuals with an active substance use disorder were also excluded. The study further excluded individuals with uncontrolled organic pathology and those who had undergone any recent changes in their pharmacological treatment regimen within the last four weeks, excluding benzodiazepines. Finally, individuals who were unable to provide informed consent were not eligible to participate in the study. Once identified and enrolled the 25 participants, they were randomly assigned to either the treatment group (n=13) that received the rTMS treatment, or the control group (n=12) that received no treatment with rTMS. All participants maintained their pre-existing pharmacological treatment regimens during the study. These included a variety of antidepressant classes, such as SSRIs (n=6), SNRIs (n=9), TCAs (n=3), NARIs (n=1), SMS (n=2), NASSAs (n=2), as well as therapies including mood stabilizers (n=1) and substituted benzamides (n=1). The distribution of these treatments across the two groups is detailed in **Table 1**.

**Table 1:** Main demographic and clinical characteristics of the sample.

Variable	rTMS group	Control group	Total
General demographics			
Participants	13	12	25
Age, years (SD)	57.9 (9,0)	64.8 (9.3)	61.2 (9.58)
Sex ratio (female/male)	10/3	6/6	16/9
Highest educational attainment			
Primary School	1	5	6
Middle School	7	3	10
High School	3	1	4
University Degree	2	3	5
Occupational status			
Occupied	4	1	5
Unoccupied	2	1	3
Housewife/housemaker	2	2	4
Retired	5	8	13
Civil status			
Single	4	0	4
Married	6	10	16
Divorced/Separated	0	2	2
Widowed	3	0	3
Clinical data			
Major Depressive Disorder	13	12	25
TRD (Stage II Thase-Rush model) assessment	13	12	25
Antidepressant Class Distribution:			
SSRI	3	3	6
SNRI	2	7	9
TCA	3	0	3
NRI	1	0	1
SMS	1	1	2
NASSA	1	1	2
Mood Stabilizer	1	0	1
Substituted Benzamide	1	0	1

TRD, Treatment Resistant Depression; SSRI, Selective serotonin reuptake inhibitors; SNRI, serotonin–norepinephrine reuptake inhibitors; TCA, tricyclic antidepressants; NRI, norepinephrine reuptake inhibitor; SMS, serotonin modulator and stimulator; NASSA, noradrenergic and specific serotonergic antidepressants.

### rTMS stimulation protocol

Participants from the treatment group (N=13) were subjected to an active rTMS stimulation protocol. In our study, we utilized a MagVenture MagPro R30 device. Stimulation was delivered using a figure-of-eight coil, model Cool D-B80 (dB/dt 31kT/s), positioned over the left dorsolateral prefrontal cortex (DLPFC). The coil was oriented with the handle pointing backward and angled approximately 45 degrees laterally from the midline, in accordance with standard guidelines for DLPFC localization. The protocol was designed and executed according to the guidelines established by the specialized operators at the facility.

Before the commencement of rTMS, the resting motor threshold (MT) for each participant was determined and stimulation site determined at 6 cm anterior to this area. The MT is specific to each patient and, although relatively stable, can vary over time (26). MT determination involved locating the “hot spot” for the right abductor pollicis brevis (APB) muscle. Muscle responses were monitored through visual observation of muscle contractions, without the use of surface electromyography (sEMG). To identify the optimal stimulation site, we began with suprathreshold intensities, systematically adjusting the coil position to locate the area that produced the largest and most consistent motor-evoked potentials (MEPs) from the APB. Resting MT was defined as the minimum stimulation intensity capable of eliciting at least 5 visible muscle movements out of 10 trials at rest. Determining the MT served two primary purposes: first, to identify the location of the hand area on the motor cortex, and second, to establish the minimum stimulation level required to elicit a consistent thumb contraction. The MT was assessed on the left hemisphere, as the subsequent rTMS treatments were applied to the left dorsolateral prefrontal cortex (DLPFC). Given the relative stability of the MT, it was typically verified every 1-2 weeks.

The rTMS treatment protocol included 20 sessions administered over 4 weeks and involved the following parameters:

Frequency: 10 Hz
Pulses per train: 40 pulses (with 4 seconds of stimulation at 10 pulses per second)
Interval between trains: 26 seconds (resulting in a total session duration of 35 minutes)
Number of trains per session: 72 trains (totaling 2880 pulses per session)
Stimulation intensity: 100% of the resting motor threshold (MT)

### Clinical and cognitive assessments

Clinical assessments were conducted at baseline one week before the treatment (T_0_) and one week after the completion of the rTMS treatment (T_1_). Depression severity was assessed by certified professionals using the Hamilton Depression Rating Scale (HAM-D 21) (24) and the Clinical Global Impression Scale (CGI) (27). The HAM-D was chosen especially because of its efficacy and sensitivity in capturing changes in patients’ depressive conditions. Cognitive performance was evaluated using a battery of tests, including the Mini-Mental State Examination (MMSE), Trail-Making Test (TMT), Digit Span Test (DST), and Verbal Fluency Test, from the Wechsler battery. These tests were selected as a means to provide a short but comprehensive evaluation of patients’ cognitive functions, which are known to be strongly associated with depressive symptomatology (16).

### BDNF sampling and analysis

Blood samples were collected from participants at two timepoints, at baseline (T_0_) and one week after the 4-week rTMS treatment (T1), between 8:00 and 10:00 a.m., to measure serum BDNF levels. Samples were stored at adequate temperature and conditions, then processed using the BDNF ELISA Kit (Booster Immunoleader Biological Technology Co., Ltd.), which employs sandwich ELISA technology for the quantitative detection of BDNF. Serum samples were allowed to clot, and centrifuged, and the supernatant was stored at −20°C for analysis. BDNF levels were measured per the manufacturer’s protocol.

### Statistical analysis

Statistical analyses were performed using RStudio (28) and Jupyter Notebook softwares. All regression models were fitted using “lme4” package (29). The first analysis was aimed at identifying the mean difference between T_0_ and T_1,_ and capturing the difference between treatment and control group, considering the statistical interaction of group x timepoint, to assess the impact of rTMS. We applied mixed ANOVA models on the scores of HAM-D, CGI, MMSE, Digit Span Test, Verbal Fluency Test (and its categorical sub-component), and on BDNF levels. The second part of the analysis aimed at testing our hypothesis of LSA being a moderator for clinical outcomes with rTMS treatment, regarding cognitive functioning. In this sense, our hypothesis was that LSA might have a negative effect on cognitive improvements that can be obtained with rTMS. Thus we conducted a series of mixed-effect linear regression models (MLMR), adjusting for sex and age, and proceeded with a Bonferroni-Holm correction for multiple comparisons (30). MLRM analysis was chosen particularly because it allows the observation of the effects and interaction of multiple independent variables on a dependent variable while considering repeated measures across participants (31). The third analysis was aimed at testing the hypothesis of BDNF levels at baseline (T_0_) being able to be a clinical predictor of positive outcomes of treatment with rTMS in TRD; for this goal we ran a simple linear regression model to evaluate the association between the BDNF at baseline (T_0_) and the improvement of the depressive symptoms (HAM-D, CGI) at T_1_.

## Results

### Sample characteristics and protocol

The study included 25 outpatients (mean age = 61.2 years, SD = 9.58) diagnosed with MDD according to DSM-IV-TR criteria, and TRD, recruited from the clinic. The sample was composed of 16 females (64%) and 9 males (36%). The gender ratio for this sample reflects the general population in both (a) depression being more common in female individuals (32) and (b) TRD being more common in female individuals (33). The two groups were formed by randomly assigning participants to treatment and controls (N=13, N=12). Most of the participants had a level of formal education of middle-school or lower (64%), possibly due to the higher mean age of participants. In fact, most participants were retired at the time of the testing (60%). Sixty percent of the participants indicated to have a married civil status. These characteristics are summarized in **Table 1**.

### rTMS impact on depressive symptoms, cognition, and BDNF levels

To assess the impact of rTMS treatment on depressive symptoms, cognitive performance, and BDNF levels in TRD, we performed a series of mixed ANOVA analyses, comparing pre- and post-treatment measurements, using a between-within interaction approach. We first ran a Shapiro-Wilk test to assess the normality of the data distribution, and the results indicated a deviation from normality, which justified the use of the non-parametric Wilcoxon test for repeated measurements of Verbal Fluency scores.

**Figure 1:**
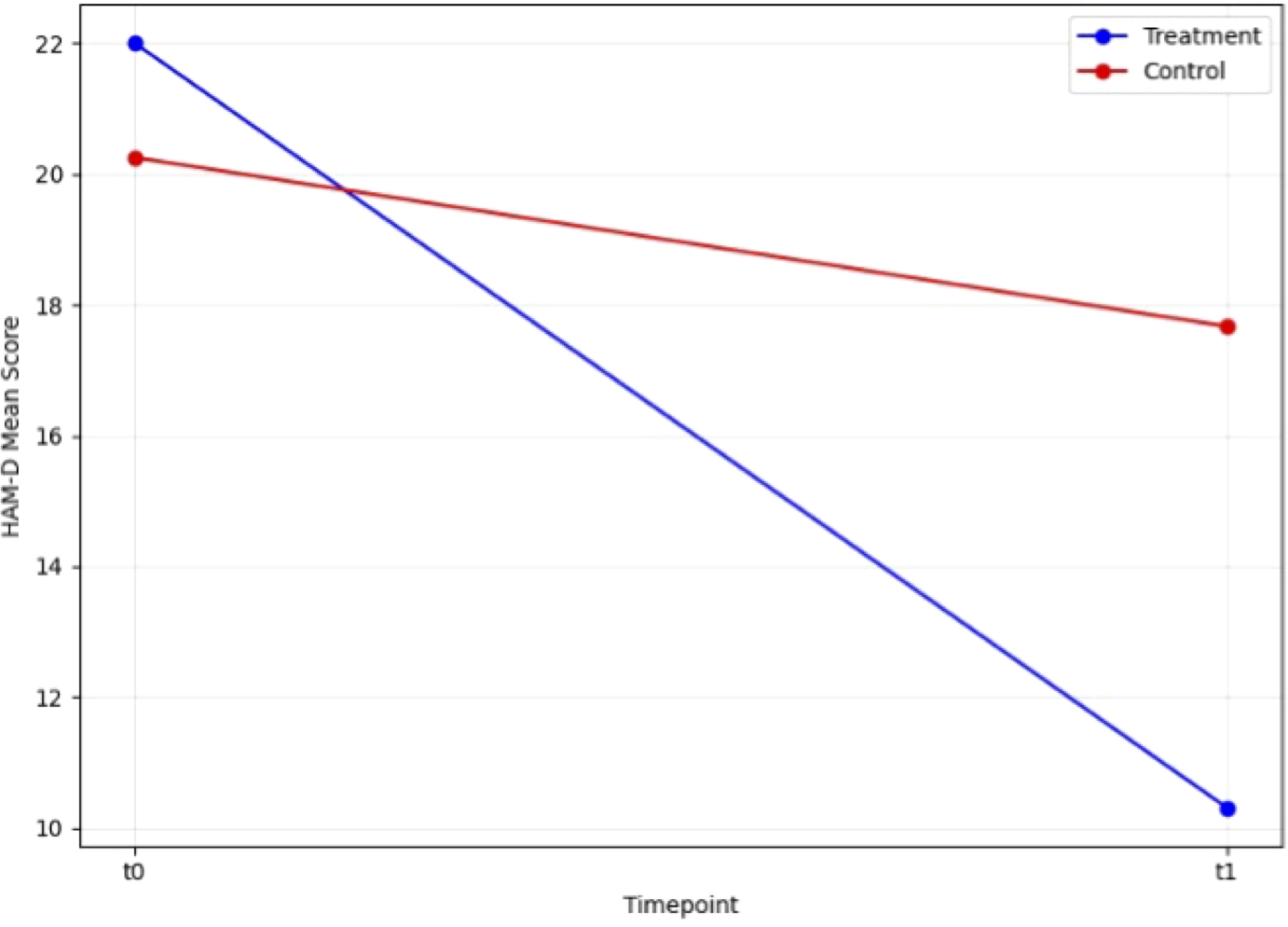
Change in HAM-D scale score during open label trial with rTMS between baseline and endpoint.

**Figure 2:**
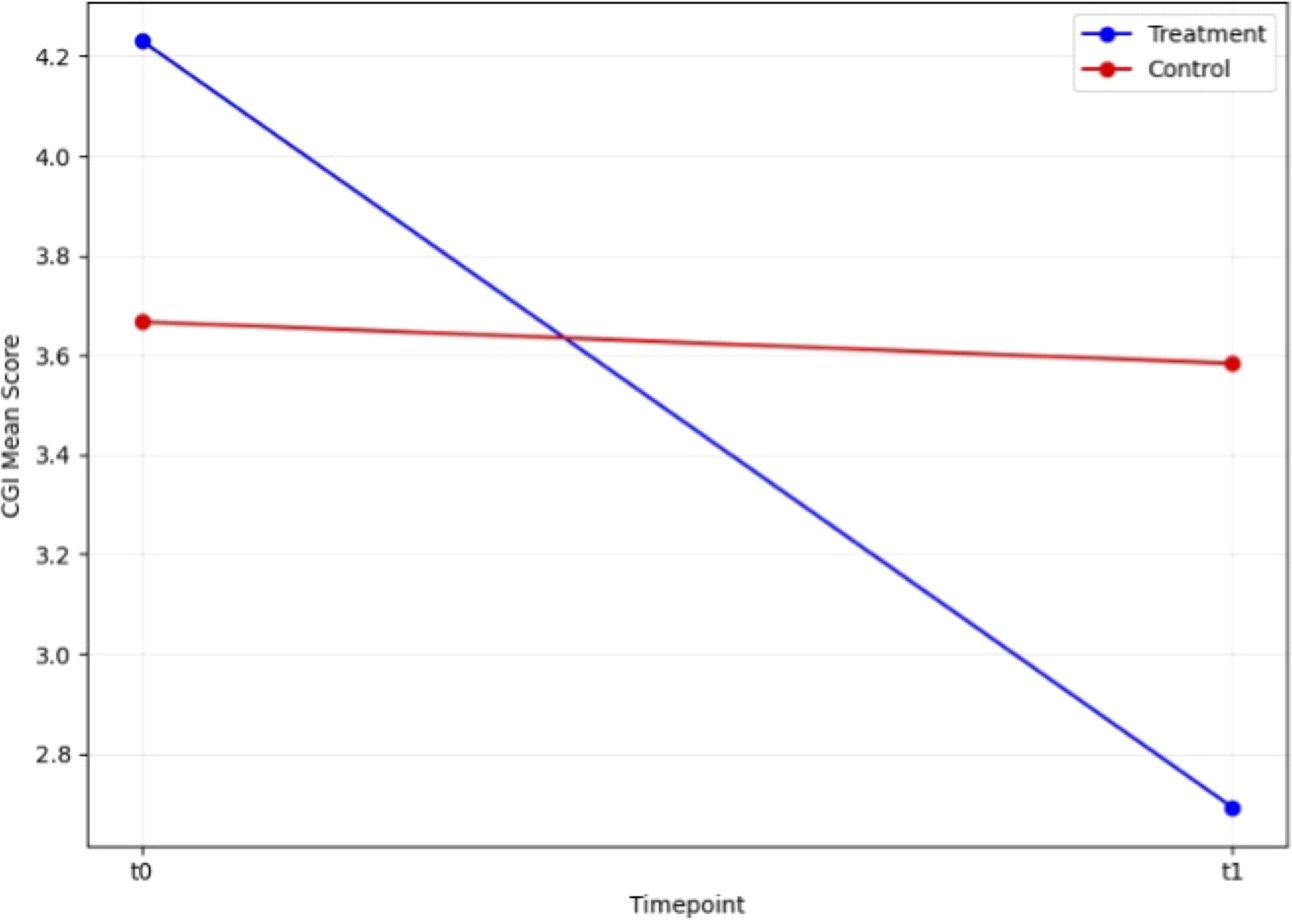
Change in CGI scale score during open label trial with rTMS between baseline and endpoint.

We first conducted paired t-tests to evaluate the efficacy of rTMS in the two groups. In the control group, the t-test revealed no significant change in HAM-D scores between t0 and t1 (T = 1.44, p=0.177). In contrast, the treatment group demonstrated a highly significant reduction in HAM-D scores from t0 to t1 (T = 6.58, p<0.001). Similarly, CGI scores in the control group showed no significant change between t0 and t1 (T = 0.43, p=0.674), while the treatment group exhibited a significant improvement (T = 6.32, p<0.001). These findings highlight the strong effect of rTMS on depressive symptoms in the treatment group, while the control group did not show notable changes (**Figures 1**, **2**).

A mixed ANOVA (**Table 2**) was conducted to examine the effects of group (treatment *vs*. control) and timepoint (pre-treatment *vs*. post-treatment) particularly with a focus on the interaction effect (group AND timepoint) on BDNF levels, HAM-D scores, CGI scores, MMSE scores, Digit Span scores, and Categorical Verbal Fluency scores. Additionally, a Wilcoxon signed-rank test was performed for Verbal Fluency scores. For HAM-D scores, a significant main effect of timepoint was observed (F(1, 23) = 16.98, p < 0.001, η² = 0.42), indicating changes in depression severity from pre- to post-treatment. Additionally, a significant interaction between group and timepoint was found (F(1, 23) = 5.32, p = 0.03, η² = 0.19). For CGI scores, there was a significant main effect of timepoint (F(1, 23) = 28.68, p < 0.001, η² = 0.55), and a significant interaction between group and timepoint (F(1, 23) = 21.48, p < 0.001, η² = 0.48). For MMSE scores, a significant interaction between group and timepoint was observed (F(1, 23) = 18.08, p < 0.001, η² = 0.44). For Digit Span scores, there was a significant main effect of timepoint (F(1, 23) = 4.98, p = 0.04, η² = 0.18), and a significant interaction between group and timepoint (F(1, 23) = 6.00, p = 0.02, η² = 0.21). After applying a Wilcoxon signed-rank test for Verbal Fluency, no significant differences were found between pre- and post-treatment scores, W = 77.50, p = 0.07.

**Table 2:** Results of mixed ANOVA analysis.

Source	Variable	SS	DF1	DF2	MS	F	p-unc	np2
BDNF	group	0.12	1	23	0.12	0.82	0.37	0.03
BDNF	timepoint	0.10	1	23	0.10	3.85	0.06	0.14
BDNF	Interaction	0.02	1	23	0.02	0.77	0.39	0.03
HAM-D	group	0.91	1	23	0.91	2.87	0.10	0.11
HAM-D	timepoint	4.95	1	23	4.95	16.98	**<0.001**	0.42
HAM-D	Interaction	1.55	1	23	1.55	5.32	**0.03**	0.19
CGI	group	0.33	1	23	0.33	0.49	0.49	0.02
CGI	timepoint	8.82	1	23	8.82	28.68	**<0.001**	0.55
CGI	Interaction	6.61	1	23	6.61	21.48	**<0.001**	0.48
MMSE	group	12.31	1	23	12.31	0.74	0.40	0.03
MMSE	timepoint	8.49	1	23	8.49	3.44	0.08	0.13
MMSE	Interaction	44.58	1	23	44.58	18.08	**<0.001**	0.44
DS	group	1.88	1	23	1.88	0.08	0.78	0.00
DS	timepoint	38.72	1	23	38.72	4.98	**0.04**	0.18
DS	Interaction	46.62	1	23	46.62	6.00	**0.02**	0.21
CAT_VF	group	94.38	1	23	94.38	0.44	0.51	0.02
CAT_VF	timepoint	50.00	1	23	50.00	1.42	0.25	0.06
CAT_VF	Interaction	25.96	1	23	25.96	0.74	0.40	0.03
VF (Wilcoxon)	W-Stat	77.50					0.07	

Significant associations (p < 0.05) are highlighted in bold. HAM-D, Hamilton Depression Rating Scale; CGI, Clinical Global Impression Scale; MMSE, Mini-Mental State Examination; DS, Digit Span Test; (CAT) VF, (Categorical) Verbal Fluency sub-test.

### Association between LSA and cognitive performance

To address the second research question, we examined the possible moderating effect of LSA on clinical outcomes with rTMS treatment. Mixed-effects linear models were used to examine the interaction effects of clinical outcome variables, group, timepoint and LSA. Two types of models were evaluated: one unadjusted and one adjusted for gender and age. **Table 3** presents the regression coefficients, z-values, and p-values for both the unadjusted and adjusted models. In the unadjusted models, a significant three-way interaction effect was found for Verbal Fluency, β = -12.752, z = -2.235, p = 0.025, suggesting an association between LSA and changes in verbal fluency over time and across groups. No significant three-way interaction effects were found for other variables. In the adjusted models, which accounted for sex and age, the interaction effect for BDNF became significant, β = 0.313, z = 2.917, p = 0.004, while the effect for MMSE also reached significance, β = 2.250, z = 1.965, p = 0.049. The adjusted model for Digit Span approached significance, β = 2.754, z = 1.916, p = 0.055. Notably, the significant interaction effect for Verbal Fluency in the unadjusted model was not sustained in the adjusted model (β = 6.433, z = 1.183, p = 0.237), suggesting that demographic factors may play a role in this association. We also applied the Bonferroni-Holm correction for multiple p-values, to mitigate the risk of type I error due to multiple testing. However, after applying the Bonferroni correction for multiple comparisons, no associations remained statistically significant across any of the outcome variables.

**Table 3:** Results of mixed effects linear models.

	Unadjusted Models				Adjusted for gender and age			
	Coef.	Std.Err.	Z	P>|z|	Coef.	Std.Err.	z	P>|z|
**BDNF**	-0.025	0.206	-0.121	0.904	0.313	0.107	2.917	**0.004**
**HAMD**	0.904	0.606	1.493	0.136	0.050	0.109	0.459	0.646
**CGI**	1.086	0.683	1.589	0.112	-0.140	0.283	-0.497	0.619
**MMSE**	2.806	1.904	1.474	0.141	2.250	1.145	1.965	**0.049**
**DS**	1.429	3.371	0.424	0.672	2.754	1.437	1.916	0.055
**VF**	-12.752	5.706	-2.235	**0.025**	6.433	5.439	1.183	0.237
**CAT_VF**	-11.819	7.277	-1.624	0.104	4.142	4.264	0.972	0.331

Significant associations (p < 0.05) are in bold. HAM-D, Hamilton Depression Rating Scale; CGI, Clinical Global Impression Scale; MMSE, Mini-Mental State Examination; DS, Digit Span Test; (CAT) VF, (Categorical) Verbal Fluency sub-test.

### BDNF as a predictor of treatment outcome

To investigate whether baseline serum BDNF levels at T0 could predict treatment outcomes in depressive symptom reduction following rTMS, a simple linear regression analysis was conducted with baseline BDNF as the predictor and post-treatment HAM-D scores as the outcome variable.

The regression model showed no significant association between baseline BDNF levels and the improvement in depressive symptoms as measured by HAM-D scores at the end of the rTMS treatment (F(1, 23) = 0.174, p = 0.680, R² = 0.008). The regression coefficient for baseline BDNF levels was β = 0.0889 (95% CI: [-0.352, 0.530]), indicating that BDNF baseline levels did not significantly predict reductions in depressive symptoms post-treatment with rTMS in our sample.

The constant (intercept) of the model, however, was significant, β = 11.98, p = 0.018, suggesting that baseline depressive symptom severity had an impact on post-treatment outcomes, independent of BDNF levels. To ensure the assumptions of linear regression were met, diagnostic checks were performed. The normality of residuals was verified using the Shapiro-Wilk test, with results indicating that the residuals were normally distributed (W = 0.970, p = 0.651). Additionally, the Breusch-Pagan test for homoscedasticity did not reveal any violations of the assumption of equal variance (χ² = 0.146, p = 0.702).

## Discussion

In this study, we aimed to assess the effects of rTMS on patients with TRD, focusing on depressive symptomatology, cognitive functioning, serum BDNF levels. Additionally, we examined the role of LSA as a potential moderator of these outcomes, and the hypothesis of serum BDNF baseline levels as predictor of clinical outcomes in depressive symptoms.

Our findings indicate that rTMS was effective in reducing depressive symptoms among patients with TRD. A significant reduction was observed in both HAM-D and CGI scores in treatment group compared to controls, supporting rTMS as a viable second-line intervention for TRD. These results are consistent with existing literature, which suggests that rTMS is more effective than sham procedures for TRD patients. The reduction in depressive symptom severity aligns with recent studies validating rTMS’s clinical utility in alleviating depressive symptoms (34). It is important to note that while rTMS led to significant reductions in depressive symptoms, the lack of a sham procedure for the control group limits the ability to determine whether improvements were solely due to rTMS or influenced by other factors, especially the placebo effect that plays a fundamental role in rTMS (35, 36).

Regarding cognitive performance, significant improvements were observed in MMSE and Digit Span scores in the treatment group compared to controls, indicating potential cognitive benefits associated with rTMS. Although no significant effects were detected in Categorical Verbal Fluency or Verbal Fluency after adjusting for multiple comparisons, the trends observed in MMSE and DS scores suggest that rTMS may positively impact certain cognitive domains, particularly working memory and global cognitive function. These findings partially support recent meta-analyses that have shown small but consistent cognitive improvements associated with rTMS in TRD patients (14, 15). However, further research is warranted to better understand the specific cognitive domains that may benefit most from rTMS in this population.

In contrast to our initial hypotheses, no significant changes were found in serum BDNF levels pre- and post-rTMS treatment. This finding contrasts with prior studies that reported both increases and decreases in BDNF following rTMS, highlighting the complex and variable nature of neuroplastic responses to brain stimulation (8, 16). The absence of an increase in serum BDNF levels following rTMS may be explained by several mechanisms. First, if rTMS promotes neuroplasticity and elevates BDNF demand in targeted brain regions, it is plausible that more BDNF is utilized by neurons locally, leading to a reduction in the peripheral BDNF available in the bloodstream. This potential transfer and utilization within the brain may contribute to lower circulating levels. Additionally, it is known that a substantial portion of serum BDNF originates from platelets. Given that rTMS can reduce stress and inflammation in patients, it may also reduce platelet activation, indirectly lowering serum BDNF levels. This effect on platelet dynamics may reflect a systemic adjustment rather than a true decline in neuroplasticity.

Moreover, it is well established that BDNF regulation is influenced by several hormonal pathways (35), including those involving cortisol, estrogen, and thyroid hormones. Cortisol, elevated during chronic stress, has been shown to suppress BDNF expression (36), whereas estrogens are known to enhance BDNF synthesis and support neuroplasticity (37). Given the relationship between endocrine function and neurotrophic factors, it is possible that interindividual variability in hormonal profiles contributed to the heterogeneous effects observed in BDNF response to rTMS. Future studies incorporating hormonal assessments may help clarify these interactions and refine our understanding of BDNF as a biomarker of neuroplasticity in TRD.

Finally, it is possible that neuroplastic changes induced by rTMS trigger a feedback mechanism that downregulates peripheral BDNF production. In this case, the brain may detect increased neuroplastic activity and adjust BDNF synthesis accordingly as a compensatory response. These results, overall, suggest that rTMS significantly reduces depressive symptoms, with promising trends in cognitive performance although failing to significantly affect serum BDNF.

### Lifetime suicide attempts as a predictor of clinical outcomes of rTMS

We further explored the impact of rTMS by hypothesizing a moderator effect of LSA on clinical and cognitive outcomes. MLMR were employed to account for repeated measures at two time-points and adjust for within-subject variability. In our analyses, we investigated whether LSA status (yes/no, binary) moderated outcomes across our symptomatic (HAM-D, CGI), cognitive (MMSE, VF, CAT_VF, DS), and biological (BDNF) measures. Ultimately, LSA did not emerge as a significant predictor of cognitive or clinical outcomes after applying Bonferroni correction for multiple comparisons. However, a significant interaction was observed between LSA and cognitive performance in the unadjusted model for VF, which did not persist after adjusting for sex and age. This suggests that while LSA may influence cognitive function, in our analysis there was no significant difference between groups, from pre to post treatment with rTMS, accounted by LSA. This association is even less robust when considering potential demographic confounders such as gender and age. These findings align with the broader literature, which has shown mixed results regarding the influence of suicidality on cognitive decline and indicates that other variables may mediate the relationship between LSA and cognitive outcomes in TRD (38, 39).

Lastly, our third research question, which focused on determining whether baseline serum BDNF could serve as a valid predictor for treatment outcomes with rTMS in TRD, yielded a negative result. The linear regression analysis confirmed that BDNF levels at baseline were not able to predict higher results in the time after the treatment, nor the changes in the clinical scores of HAM-D (T_1_-T_0_). In the scientific literature it is still not clearly understood if BDNF baseline levels can be adopted as a predictor of positive outcomes (40) and our results go against this potential hypothesis. BDNF levels were not predictive of treatment response in terms of depressive symptom reduction, nor remission, but other factors may be more critical in influencing outcomes and further research is needed in this proclivity to better understand the mechanisms of response to rTMS in TRD. Overall, our research focused on a pivotal concept such as treatment resistance, producing results that confirm the good fit of rTMS for reducing depressive symptoms, improving cognitive functions, reinforcing the association between suicidality (LSA) and lower cognitive functioning, while failing to elect BDNF levels as a clinical outcome predictor.

### Limitations

Our results should be interpreted considering several limitations. First, it is important to note that all patients treated with rTMS received no modifications to their pharmacological treatment, at the time of the treatment.

A major limitation of our study is the lack of a sham rTMS procedure for the control group, which prevents us from fully disentangling the effects of rTMS from non-specific factors such as expectancy effects. Although similar studies have shown efficacy in alleviating depressive symptoms (41), and have been included in further meta-analyses (42), the results of this study have to be interpreted in light of this methodological issue. Previous studies have shown that sham rTMS can lead to improvements in depressive symptoms, raising concerns about placebo contributions in clinical trials (43). Furthermore, the intensive nature of the rTMS protocol in the treatment group, contrasted with the awareness of not receiving stimulation in the control group, may have introduced additional bias (43, 44). Future studies should incorporate a rigorous sham control condition to better isolate the direct effects of rTMS from expectancy-driven responses.

Further considerations regarding this confounding aspect should be considered as a limitation as well. Being an open-label study, the evaluators were not blinded to the treatment assignment, which may have introduced a potential bias in the assessment of clinical and cognitive outcomes. Further, this study has the inherent limitation of being a pilot analysis. Thus, the findings should be interpreted with caution, acknowledging the limitations imposed by the sample size. The rTMS protocol employed in this study adhered to established scientific standards, with a total of 20 sessions administered over 4 weeks at an intensity of 100% of the resting motor threshold. These parameters are consistent with the current consensus in the literature, which highlights their efficacy in treatment-resistant depression (45). Moreover, a potential limitation is the use of the traditional 5 cm rule for coil positioning over the left DLPFC, which may be less accurate than neuronavigation-based methods. Future studies could benefit from employing the BeamF3 method, which has been shown to improve localization accuracy and individual anatomical targeting (46). However, it is worth noting that some studies suggest that a higher number of sessions, extended beyond 4 weeks, or a slightly increased stimulation intensity may yield additional benefits in certain patient populations (45).

Another relevant limitation is the lack of control for hormonal variations, particularly in female participants, predominant in the active group (10 out of 13 females). Given that estrogen and progesterone modulate BDNF expression and neuroplasticity (35), and that the majority of our sample consisted of perimenopausal or postmenopausal women, hormonal fluctuations may have influenced BDNF results.

Several limitations are linked to the use of peripheral BDNF levels as a biological variable in this instance. We did not collect plasma samples to assess BDNF concentration, which could be seen as a limitation of this study. A previous systematic review suggested that plasma BDNF, as opposed to serum, showed significantly lower levels in individuals with LSA (47). However, the interpretation of these findings should take into account the known instability of plasma BDNF measurements and the relatively low test-retest reliability over extended periods, which has led some authors to recommend serum assessments for more consistent results (48). Also, BDNF levels may be influenced by platelet activation, as platelets are a major source of circulating BDNF. Since rTMS may indirectly reduce platelet activation through stress and inflammation reduction, it’s possible that changes in BDNF levels could reflect fluctuations in platelet activity rather than solely central neuroplastic changes. Future studies should consider platelet activation as a potential confounding factor when interpreting peripheral BDNF levels (48). Besides that, the relationship between central and peripheral BDNF levels remains unclear, with studies offering both supporting and opposing evidence regarding the use of peripheral BDNF as a reliable biomarker for mental disorders (12). Furthermore, another limitation of our study is the inability of ELISA kits we used to differentiate between pro-BDNF and mature BDNF. Unlike mature BDNF, pro-BDNF is involved in processes such as inducing apoptosis and reducing dendritic spines, which can contribute to long-term depression (49). Differentiating between these isoforms is crucial, as they may exert opposing effects. Future research should aim to utilize newly developed specific assays for mBDNF and pro-BDNF (50) to better investigate these associations. The cognitive assessments employed, namely the Mini-Mental State Examination (MMSE) and Digit Span tests, may lack the sensitivity to detect subtle executive dysfunctions in TRD patients. Future studies should consider incorporating more specific instruments, such as the Montreal Cognitive Assessment (MoCA), the Digit Symbol Substitution Test (DSST), or the Stroop Test, to capture nuanced cognitive changes. Acknowledging that LSA was analyzed as a binary variable (yes/no), future studies could benefit from using more granular measures of suicidality, such as the total number of suicide attempts or thresholds based on their distribution (e.g., median split). These approaches may better capture the nuanced role of suicidality in moderating cognitive or clinical outcomes. In future studies, a larger and more homogeneous sample will be essential to address these limitations. The relatively small sample size limited the ability to perform subgroup analyses and constrained the inclusion of covariates in the MLRM models to avoid overfitting. Moreover, despite using a longitudinal design and applying MLRM, it remains possible that some time-varying variables were not fully captured by our analysis.

## Conclusions

This study aimed to elucidate the effects of rTMS on depressive symptoms, cognitive function, and BDNF levels in TRD, while also exploring the influence of LSA on these outcomes. Our findings indicate that rTMS exerts a significant positive impact on reducing the severity of depressive symptoms, evidenced by significant reductions in both HAM-D and CGI scores. This supports the wide body of literature that positions rTMS as a reliable intervention for individuals unresponsive to conventional antidepressant treatments. In terms of cognitive outcomes, statistical significance was achieved in MMSE and Digit Span Test scores in the treatment group compared to controls, indicating a positive effect of rTMS. Other tests scores, such as Verbal Fluency Test increased marginally but did not reach significance, indicating a trend towards improvement in cognitive tasks and working memory. The implications of these findings are that rTMS might enhance certain cognitive functions, albeit with smaller and more variable effects compared to its impact on mood. Contrary to our expectations, BDNF levels did not increase significantly post-treatment in the treatment group. This finding highlights the complexity of BDNF as a biomarker in depression and response to brain stimulation, which remains inconsistent across studies. Moreover, our analysis suggested that a history of suicide attempts is not significantly linked to less improved cognitive performance changes in rTMS treatment of TRD, as the initial significance in Verbal Fluency Test scores did not withstand corrections. Baseline BDNF levels also failed to predict treatment outcomes, aligning with mixed findings in the literature. These results emphasize the need in future studies for larger samples, refined biomarkers, and advanced techniques like TBS or fMRI-guided rTMS to better explore the interplay between neuroplasticity, depressive symptoms, and cognitive outcomes.

## Data Availability

The raw data supporting the conclusions of this article will be made available by the authors, without undue reservation.

## In memoriam

This work is dedicated to the loving memory of Dr. Maria Novella Iaselli, esteemed colleague and re-searcher, prematurely deceased.
